# Nearly Complete Genome Sequence of a Novel Phlebovirus-Like Virus Detected in a Human Plasma Sample by High-Throughput Sequencing

**DOI:** 10.1128/MRA.00764-19

**Published:** 2019-08-29

**Authors:** Florian Laubscher, Samuel Cordey, Mary-Anne Hartley, Gael Vieille, Noémie Boillat-Blanco, Josephine Samaka, Tarsis Mlaganile, Valerie d’Acremont, Laurent Kaiser

**Affiliations:** a Laboratory of Virology, University Hospitals of Geneva, Geneva, Switzerland; b University of Geneva Medical School, Geneva, Switzerland; c Centre for Primary Care and Public Health (Unisanté), University of Lausanne, Lausanne, Switzerland; d Swiss Tropical and Public Health Institute, University of Basel, Basel, Switzerland; e Infectious Diseases Service, Lausanne University Hospital, Lausanne, Switzerland; f Ifakara Health Institute, Dar es Salaam, United Republic of Tanzania; g Geneva Centre for Emerging Viral Diseases, University Hospitals of Geneva, Geneva, Switzerland; KU Leuven

## Abstract

Here, we report a novel phlebovirus-like virus sequence detected in a plasma sample from a febrile adult patient collected in the United Republic of Tanzania in 2014. A nearly complete RNA sequence was generated by high-throughput sequencing on a HiSeq 2500 instrument and further confirmed after repeating the analysis, starting from the initial sample.

## ANNOUNCEMENT

Phleboviruses (*Bunyavirales* order, *Phenuiviridae* family) represent single-stranded RNA viruses composed of three segments (S, M, and L). According to the 10th report of the International Committee on Taxonomy of Viruses in 2018 (https://talk.ictvonline.org/taxonomy/), the *Phlebovirus* genus is classified into 10 species; its best known member is the mosquito-borne Rift Valley fever virus that infects livestock, wild animals, and humans (1). Several other phleboviruses have been described that infect humans via phlebotomine sandflies or ticks, and although frequently asymptomatic, they may trigger febrile illness or central nervous system infections (2–4).

During a high-throughput sequencing (HTS)-based investigation into the possible viral associations in adult patients with unexplained febrile illness, we detected a nearly complete RNA sequence from a novel phlebovirus-like virus in a plasma specimen collected in March 2014 in Dar es Salaam, United Republic of Tanzania. The specimen originated from an HIV-negative 43-year-old male attending the outpatient department with a 3-day history of fever, headache, and back pain and 2 days of reported pollakiuria. At the time of consultation, the patient was febrile (38.1°C), with a normal blood pressure and pulse rate. Despite extensive microbiological investigations (blood and urine cultures; rapid diagnostic tests for dengue, malaria, and typhoid fever; multiplex PCR targeting tropical causes of fever; and serologies for Epstein-Barr virus, cytomegalovirus, toxoplasmosis, and histoplasmosis), the cause of fever remained unknown.

The plasma sample was prepared for HTS analysis as previously described (i.e., centrifugation, DNase treatment, and RNA extraction by TRIzol) (5). The RNA library was prepared using the TruSeq total RNA preparation protocol (Illumina, San Diego, CA, USA) and run on the HiSeq 2500 platform (Illumina) using the 2 × 100-nucleotide read length protocol. Adapters were trimmed and quality checked using Trimmomatic (v0.33) (parameters included Illuminaclip, TruSeq3-PE-2.fa:1:30:10:4:true; MAXINFO, 40:0.3; and minlen, 36) (6). Human reads were removed by mapping reads against the human genome and transcriptome (hg38; Gencode v23) using SNAP (v1.0beta.23; command, paired with default parameters) (7). The *de novo* assembly was made using IDBA-UD (v1.1.3; parameters included pre_correction, –step 20, –mink 21, and –maxk 100) (8). Contigs were subjected to a BLAST search (BLASTX, v2.3.0+) (9) against the U-RVDBv12.2 viral database (https://rvdb-prot.pasteur.fr/). Then, reads were mapped back to the contigs using SNAP. A total of 4,059, 5,112, and 7,401 reads specific to the S, M, and L segments, respectively, were remapped (median read depths, 337, 129, and 113, respectively). One contig was obtained for each individual segment. The BLAST result showed a maximum of 31% amino acid identity (51% similarity) with otter fecal bunyavirus (GenBank accession number AIB06815) for the S segment, 27% amino acid identity (46% similarity) with Pidgey bunyavirus (GenBank accession number KX852390) for the M segment, and 31% amino acid identity (50% similarity) with Zaliv Terpenia virus (Uukuniemi phlebovirus) (GenBank accession number KF767463) for the L segment. Of note, dengue virus-specific sequences were detected but resulted from index hopping. None of the 31 RNA libraries in the same run (including two HTS whole-process controls) were found to be positive for this novel phlebovirus-like sequence. Nevertheless, such discoveries should be interpreted with caution because they could result from a punctual HTS environmental contamination (10). To this end, the analysis was repeated, starting from the initial plasma sample. The novel RNA library was run alone on a HiSeq 4000 instrument (Illumina), which confirmed the presence of this phlebovirus-like virus sequence (a total of 60,605, 80,846, and 119,580 reads specific to the S, M, and L segments, respectively).

**FIG 1:**
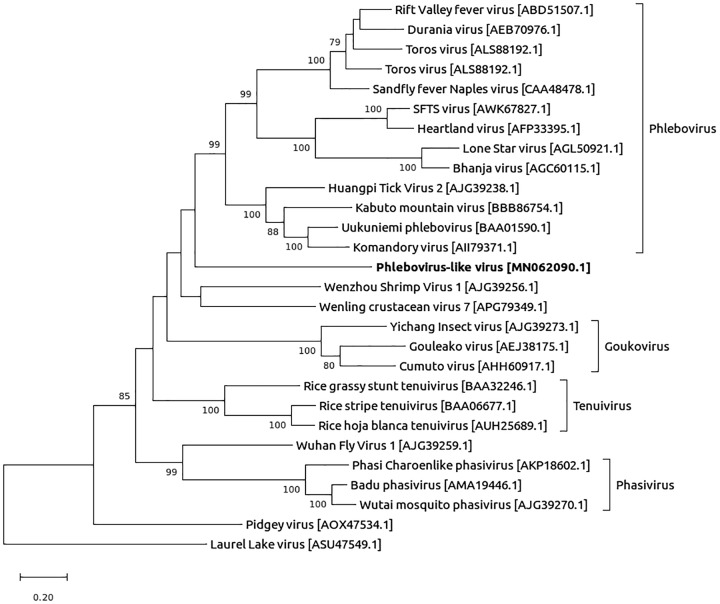
Phylogenetic tree constructed for the RNA-dependent RNA polymerase using a 500-replicate maximum likelihood method based on the LG model (11), using the Laurel Lake virus (GenBank accession number ASU47549) as an outgroup. The alignment and tree building were done using SeaView v4.7 (MUSCLE v3.8.31) and the MEGA X software, respectively.

The nearly complete virus genome sequence detected was 1,309, 3,776, and 6,474 nucleotides long for the S, M, and L segments, respectively (G+C contents of 41.0%, 41.6%, and 39.1%, respectively), covering the respective complete coding sequences. A phylogenetic analysis performed on the L segment showed that this viral RNA sequence was related to *Phlebovirus* genus sequences (Fig. 1). Although the detection of this novel phlebovirus-like sequence should be interpreted with caution (i.e., the detection of a sequence does not prove by itself a true human infection), the report of such a sequence is important since phleboviruses are known to infect humans and can trigger febrile illness or central nervous system infections.

### Data availability.

The nucleotide sequences detected from the clinical specimen collected in the United Republic of Tanzania were deposited in GenBank under the accession numbers MN062090, MN062091, and MN062092. The raw sequence data were deposited in the NCBI Sequence Read Archive under BioProject accession number PRJNA541799.
